# Association of Charlson comorbidity index with 28- and 90-day mortality in patients with severe acute kidney injury requiring CRRT: A retrospective cohort study

**DOI:** 10.1097/MD.0000000000050884

**Published:** 2026-09-25

**Authors:** Kejia Li, Yuefei Xiao, Qingyan Ma, Liqin Tang, Jing Han, Chunhong Guo, Yunyun Yang, Jing Zhang

**Affiliations:** a Department of Nephrology, Aerospace Center Hospital, Beijing, China; b Minimally Invasive Spine Surgery Department, The Second Affiliated Hospital of Guangzhou University of Chinese Medicine, Guangzhou, China.

**Keywords:** acute kidney injury, Charlson comorbidity index, continuous renal replacement therapy, mortality, prognosis

## Abstract

Severe acute kidney injury (AKI) requiring continuous renal replacement therapy (CRRT) carries high short‑term mortality. This study evaluated whether the Charlson Comorbidity Index (CCI) independently predicts 28‑ and 90‑day mortality and adds incremental prognostic value beyond established clinical variables. This retrospective study of 1115 adult patients with severe AKI treated with CRRT, CCI was computed at intensive care unit (ICU) admission and categorized (0–1, 2–3, 4–5, ≥6). Primary outcomes were 28‑ and 90‑day mortality. Multivariable Cox proportional hazards models were adjusted for demographics and treatment. Nonlinearity was explored using restricted cubic splines. Robustness was tested using propensity score matching and weighting. The 28‑ and 90‑day mortality rates were 61.3% and 71.1%. A higher CCI was associated with progressively worse survival (log-rank *P* < .001). As a continuous variable, CCI correlated with increased mortality after full adjustment (28‑day hazard ratio [HR] per point 1.07, 95% confidence interval [CI] 1.03–1.11; 90‑day HR 1.06, 95% CI 1.03–1.09; *P* < .001). CCI cases ≥6 versus 0 to 1 had higher adjusted hazards (28 day HR 1.81; 95% CI 1.40–2.33; 90‑day HR 1.66; 95% CI 1.31–2.10, *P* < .001). These findings were consistent across the sensitivity and subgroup analyses. Adding CCI to a base model improved C‑statistics (28‑day 0.759 to 0.775, *P* = .002; 90‑day 0.763 to 0.777, *P* = .017) and yielded significant net reclassification index/integrated discrimination improvement gains. In severe AKI-CRRT cases, CCI independently predicted 28‑ and 90‑day mortality and modestly improved prognostic discrimination beyond acute severity measures. Routine assessment of CCI may refine early risk stratification, helping prioritize monitoring and supportive interventions for the highest-risk patients.

## 1. Introduction

Severe acute kidney injury (AKI) that necessitates continuous renal replacement therapy (CRRT) is a frequent and devastating complication in critically ill adults, with reported in-hospital mortality rates often exceeding 40% and substantial residual morbidity among survivors.^[1–4]^ International guidelines emphasize the heterogeneity of AKI and the importance of early risk stratification to guide clinical decisions, prognostication, and resource allocation.^[5]^ Traditional severity scores (e.g., Acute Physiology and Chronic Health Evaluation [APACHE] and Sequential Organ Failure Assessment [SOFA]) capture acute physiological derangement but do not fully account for the chronic comorbidity burden, which can substantially modulate resilience to acute organ failure and influence short-term survival after CRRT initiation.^[6]^

The Charlson Comorbidity Index (CCI) is a widely validated instrument that quantifies comorbidity burden to improve mortality prediction across diverse populations.^[7,8]^ In critical care and chronic dialysis cohorts, higher comorbidity scores have been associated with worse outcomes.^[9–11]^ However, data specifically addressing the prognostic value of CCI in patients with severe AKI requiring CRRT are limited and inconsistent. Several multicenter epidemiological studies have characterized the incidence and outcomes of AKI in the intensive care unit (ICU) and documented the dominant influence of acute severity measures on early mortality^[3,12,13]^; however, few studies have systematically evaluated whether CCI provides independent prognostic information for 28- and 90-day mortality beyond acute illness scores and treatment variables (e.g., vasopressor use, mechanical ventilation (MV), and timing/intensity of CRRT). CCI is readily available upon patient admission through history-taking.^[8]^ Establishing the added predictive CCI value could facilitate bedside risk stratification using a simple, objective tool available at CRRT initiation.

Given the biological plausibility that chronic comorbidity diminishes physiological reserve and affect recovery trajectories after severe AKI, this study tested the hypothesis that CCI independently predicts 28- and 90-day mortality after adjusting for acute illness severity and therapeutic factors. High-risk patients require an early diagnosis and enhanced care. Consequently, this study examined the independent correlation between CCI score and mortality among patients with severe AKI undergoing CRRT.

## 2. Methods

### 2.1. Study population

This retrospective cohort investigation analyzed original data from a publicly accessible repository (https://datadryad.org), provided by Jung et al.^[14]^ This study was approved by the Institutional Review Board of Yonsei University Health System, Severance Hospital, and adhered to the principles of the Declaration of Helsinki (approval no. 4-2016-1073). Due to the retrospective design utilizing de-identified medical records, the Institutional Review Board granted a waiver for written patient consent. The full methodological details have been documented elsewhere.^[14]^ Furthermore, reporting adhered to the Strengthening the Reporting of Observational Studies in Epidemiology guidelines.

In total, 2391 adult patients (≥18 years) with severe AKI requiring CRRT in ICUs at National Health Insurance Service Medical Center Ilsan Hospital and Yonsei University Health System Severance Hospital, from January 2009 to September 2016, underwent initial screening for inclusion. AKI and its staging were determined in accordance with the AKI Network AKI network guidelines using both serum creatinine (SCr) and urine output criteria.^[15]^ AKI was defined as an absolute increase in SCr by ≥0.3 mg/dL (≥26.4 μmol/L) within 48 hours, or SCr levels reaching 1.5 to 2.0 times the baseline value, or urine output <0.5 mL/kg/hour for more than 6 hours.^[15]^ AKI stages were defined as follows: Stage 1: SCr elevation ≥ 0.3 mg/dL (26.4 μmol/L) or reaching 1.5 to 1.9 times the baseline level, and/or urine output < 0.5 mL/kg/hour for ≥6 hours; Stage 2: SCr elevation to 2.0 to 2.9 times the baseline level, and/or urine output <0.5 mL/kg/hour for ≥12 hours; Stage 3: SCr elevation to ≥3.0 times the baseline level or ≥4.0 mg/dL (354 μmol/L), with an acute increase of at least 0.5 mg/dL (44 μmol/L), and/or urine output <0.3 mL/kg/hour for ≥24 hours or anuria for ≥12 hours.^[15]^ Stage 2 or 3 AKI was defined as severe AKI. The exclusion criteria were as follows: stage 1 AKI, age <18 years, pregnancy or lactation, preexisting chronic kidney disease or receipt of dialysis or CRRT before enrollment, post-renal obstruction, previous kidney transplantation, extreme mean arterial pressure (MAP) or body mass index (BMI), and time to death or outliers. Therefore, 1115 patients were included in the final analysis.

### 2.2. Exposure variable

On admission, each patient’s medical history was reviewed, and the comorbidity burden was computed with CCI. The CCI assigns weighted scores to 18 comorbidity categories, following the original weights described by Charlson et al^[16]^: myocardial infarction; congestive heart failure; cerebral vascular disease; peripheral vascular disease; dementia; chronic pulmonary disease; connective tissue disease; peptic ulcer disease; mild liver disease; and diabetes mellitus (weight 1); hemiplegia; diabetes with end-organ damage; any solid tumor; leukemia; and lymphoma (weight 2); moderate-to-severe liver disease (weight 3); and metastatic solid tumor and acquired immunodeficiency syndrome (weight 6), classified according to the International Classification of Diseases, Tenth Revision (ICD-10) codes (Supplementary Table S1, Supplemental Digital Content 1). Although the original CCI appendix provides a category for moderate-to-severe renal disease, this study did not score underlying kidney disease because the cohort comprised patients with severe AKI receiving CRRT. CCI scores were categorized into 4 levels of comorbidity severity based on the patient’s comorbidity conditions: 0 to 1 points (low), 2 to 3 points (mild), 4 to 5 points (moderate), and ≥6 points (severe).^[17]^

### 2.3. Covariates

Upon ICU admission, patient demographic data and comorbidities, including age, sex, height, and weight, were obtained from the hospital information system. BMI was calculated as the weight in kilograms divided by the square of the height in meters. Laboratory measurements taken at the initiation of CRRT were used as baseline values, including albumin, hemoglobin, blood urea nitrogen (BUN), creatinine, phosphate, potassium, C-reactive protein (CRP), white blood cell, and bicarbonate levels. The estimated glomerular filtration rate (eGFR) was determined by an automated analyzer using standard methods. Disease severity was assessed using various clinical parameters: (physiologic and chronic health evaluation II [APACHE II]) score, SOFA score, MAP, MV, 2hour urine output at CRRT initiation, CRRT dose, cause of AKI (sepsis or not), and AKI network stage. Sepsis-related AKI was recorded when sepsis was documented as the etiology of AKI at the time of CRRT initiation, based on the clinical diagnoses recorded in the source dataset.

### 2.4. Outcomes

Primary outcome indicators were mortality at 28 and 90 days after CRRT initiation. The index date (time zero) was defined as the date and time of CRRT initiation. Patients were followed from time zero until death or day 28 and day 90, respectively; patients alive at the end of each window or lost to follow-up were censored at that time.

### 2.5. CRRT protocol

CRRT initiation was decided by nephrologists based on AKI development in the ICU patients.

Temporary vascular access is typically established using a catheter placed in the internal jugular, subclavian, or femoral vein to initiate CRRT. Dialyzers with membrane surface areas of 1.0 to 1.4 m^2^ were used. CRRT was initiated at a blood flow rate of 100 mL/minutes, which was increased to 150 mL/minutes. The total effluent volume was aimed at a dose of 35 mL/kg/hour for all patients. The mean duration of the first CRRT session was 40 hours.

### 2.6. Statistical analysis

We observed that fewer than 30% of the covariates had missing values. A multivariate single-imputation method for missing data based on iterative imputation was implemented using a Bayesian Ridge model as the estimator at each step of round-robin imputation.^[18]^ Categorical variables are reported as numbers (percentages). Continuous variables are presented as mean ± standard deviation. Differences between groups were compared using the Student *t* test, Mann–Whitney *U* test, or chi-square test, as appropriate. The Kaplan–Meier method and log-rank test were used to estimate the 28- and 90-day survival curves. Multivariate Cox proportional hazards models were fitted to estimate hazard ratios (HRs) and 95% confidence intervals (CIs) for the associations between the CCI and mortality outcomes. Potential covariates were selected if their inclusion altered the risk estimate by ≥10% or based on prior research and clinical relevance to the outcomes.^[19]^ Multicollinearity was evaluated by computing the variance inflation factor (VIF), where values >5 suggested multicollinearity (Supplementary Table S2, Supplemental Digital Content 2). For each analysis, 4 levels of hierarchical multivariate adjustment were examined: crude (unadjusted); model 1: adjusted for age, sex, and BMI; model 2: additionally adjusted for laboratory parameters (phosphate, creatinine, BUN, C-reactive protein , white blood cell count, hemoglobin, albumin, bicarbonate, and potassium); and model 3 (fully adjusted): model 2 plus disease severity (SOFA and APACHE II scores), MAP, MV, eGFR, 2-hour urine output at CRRT initiation, CRRT dose, AKI stage, and AKI cause. A Cox proportional hazards regression model with cubic spline functions and smooth curve fitting (restricted cubic splines) was used to address nonlinearity.

Multiple sensitivity and subgroup analyses were performed to assess the robustness of the findings. This study converted CCI into a categorical variable and calculated *P* for the trend to verify CCI results as a continuous variable and to examine the possibility of nonlinearity. Propensity score matching (PSM) was performed using 1:1 nearest-neighbor matching with a caliper width of 0.2; balance was evaluated using standardized mean differences (SMDs). The SMD and statistical significance of the observed differences were calculated using the paired *t* test for continuous covariates and the chi-squared test for categorical covariates. Furthermore, the previously listed covariates were incorporated to compute the propensity scores. These estimated scores were applied as analytical weights. Pairwise algorithmic (PA) and standardized mortality ratio weighting (SMRW) were used to construct a weighted cohort that balanced the baseline confounders^,[20]^ thereby providing a more accurate estimate of the independent association between CCI and mortality.

Diagnostic performance was assessed using receiver operating characteristic (ROC) analysis, with discrimination quantified by the area under the curve ;(C-statistic) for the Cox model mortality prediction. area under the ROC curve were compared using the DeLong test. The incremental prognostic value of the CCI over established risk factors was evaluated using the net reclassification index (NRI) and Integrated Discrimination Improvement (IDI).

Analyses were performed using R 4.2.2 (http://www.Rproject.org; The R Foundation, Vienna, Austria) and Free Statistics software (version 2.3; Beijing Free Clinical Medical Technology Co., Ltd, Beijing, China). *P* < .05 was considered to indicate a statistically significant result.

## 3. Results

### 3.1. Patient characteristics

In total, 1115 patients with severe AKI requiring CRRT were included in the final analysis (Fig. 1). Patients were stratified into 4 groups based on their CCI scores: 0 to 1 (n = 261), 2 to 3 (n = 462), 4 to 5 (n = 216), and ≥ 6 (n = 176). According to Table 1, the overall cohort had a mean age of 63.4 ± 14.3 years, with 685 (61.4%) male patients and an average BMI of 23.9 ± 4.4 kg/m^2^. No significant differences in age, sex distribution, or BMI were observed across CCI categories (*P* > .05 for all). Laboratory parameters revealed several significant trends. Hemoglobin levels demonstrated a progressive decline with increasing CCI (*P* < .001). BUN and SCr were significantly elevated in higher CCI groups (*P* = .001). Clinical measures indicated that the use of MV was highest in the lowest CCI group (83.1%) and decreased moderately to 71.6% in the ≥6 group (*P* = .011). EGFR declined as the CCI increased (*P* = .006). The APACHE II scores remained comparable among groups (*P* = .856).

**Table 1:** Baseline characteristics of patients.

Variables	Overall	CCI				*P* Value
		0–1	2–3	4–5	≥6	
Number	1115	261	462	216	176	
Age(yr)	63.4 ± 14.3	63.0 ± 16.0	63.7 ± 14.0	64.4 ± 14.2	61.8 ± 12.5	.278
Male, n (%)	685 (61.4)	151 (57.9)	275 (59.5)	144 (66.7)	115 (65.3)	.124
BMI (kg/m^2^)	23.9 ± 4.4	23.8 ± 4.5	23.8 ± 4.3	24.1 ± 4.7	23.9 ± 4.0	.875
Laboratory data						
Potassium (mEq/L)	4.7 ± 1.1	4.8 ± 1.1	4.6 ± 1.0	4.7 ± 1.1	4.7 ± 1.2	.169
Bicarbonate (mEq/L)	16.9 ± 5.8	16.6 ± 5.2	17.3 ± 5.8	17.2 ± 6.1	16.1 ± 5.9	.079
Phosphate (mg/dL)	5.7 ± 2.4	5.8 ± 3.0	5.6 ± 2.2	5.8 ± 2.2	5.9 ± 2.1	.450
White blood cell (×10^3^µL)	11.7 (6.6, 18.7)	11.8 (7.1, 17.0)	11.6 (6.6, 19.2)	11.8 (7.6, 17.3)	11.7 (5.2, 19.8)	.930
Haemoglobin (g/dL)	9.7 ± 2.2	10.2 ± 2.3	9.6 ± 2.2	9.4 ± 2.0	9.2 ± 2.1	< .001
BUN (mg/dL)	55.8 ± 30.0	51.4 ± 31.6	54.6 ± 28.1	61.5 ± 31.4	58.9 ± 29.7	.001
Creatinine (mg/dL)	2.7 ± 1.6	2.5 ± 1.6	2.7 ± 1.5	3.1 ± 2.0	2.9 ± 1.4	.001
Albumin (g/dL)	2.6 ± 0.6	2.6 ± 0.7	2.6 ± 0.5	2.7 ± 0.5	2.6 ± 0.6	.224
CRP (mg/L)	75.7 (20.3, 169.8)	59.8 (16.8, 157.1)	90.1 (23.0, 179.0)	51.9 (19.5, 144.6)	100.8 (25.0, 192.5)	.022
Clinical measures						
MAP (mm Hg)	77.8 ± 14.1	76.5 ± 14.1	77.9 ± 14.3	79.2 ± 14.1	77.6 ± 13.8	.215
MV, n (%)	872 (78.2)	217 (83.1)	369 (79.9)	160 (74.1)	126 (71.6)	.011
eGFR (mL/min/1.73m^2^)	26.3 (16.9, 38.9)	29.1 (19.9, 41.6)	26.8 (16.9, 39.8)	22.2 (16.1, 34.8)	24.5 (16.4, 35.8)	.006
Urine output in 2h (mL)	35.0 (5.0, 100.0)	40.0 (10.0, 110.0)	40.0 (5.0, 110.0)	23.5 (5.0, 75.0)	30.0 (2.8, 85.0)	.055
APACHE II score	27.2 ± 8.0	27.2 ± 8.1	27.3 ± 7.8	26.8 ± 8.0	27.4 ± 8.2	.856
SOFA score	12.0 ± 3.5	11.6 ± 3.0	11.9 ± 3.5	12.9 ± 3.9	12.0 ± 3.8	.001
CRRT dose (mL/kg)	36.7 ± 4.8	36.5 ± 4.6	36.9 ± 4.8	36.7 ± 4.6	36.3 ± 5.2	.408
AKI stage, n (%)						.237
Stage 2	291 (26.1)	58 (22.2)	132 (28.6)	52 (24.1)	49 (27.8)	
Stage 3	824 (73.9)	203 (77.8)	330 (71.4)	164 (75.9)	127 (72.2)	
Sepsis, n (%)	784 (70.3)	187 (71.6)	341 (73.8)	130 (60.2)	126 (71.6)	.003
Outcomes						
28-d mortality, n (%)	683 (61.3)	134 (51.3)	279 (60.4)	141 (65.3)	129 (73.3)	< .001
90-d mortality, n (%)	793 (71.1)	162 (62.1)	325 (70.3)	165 (76.4)	141 (80.1)	< .001

Data are presented as numbers (%) for categorical variables. Continuous variables are presented as mean ± standard deviation for normally distributed data or median (interquartile range) for skewed data.

AKI = acute kidney injury, APACHE = acute physiology and chronic health evaluation, BMI = body mass index, BUN = blood urea nitrogen, CCI = Charlson Comorbidity index, CRP = C-reactive protein, CRRT = continuous renal replacement therapy, eGFR = estimated glomerular filtration rate, MAP = mean arterial pressure, MV = mechanical ventilation, SOFA = sequential organ failure assessment.

**Figure 1.**
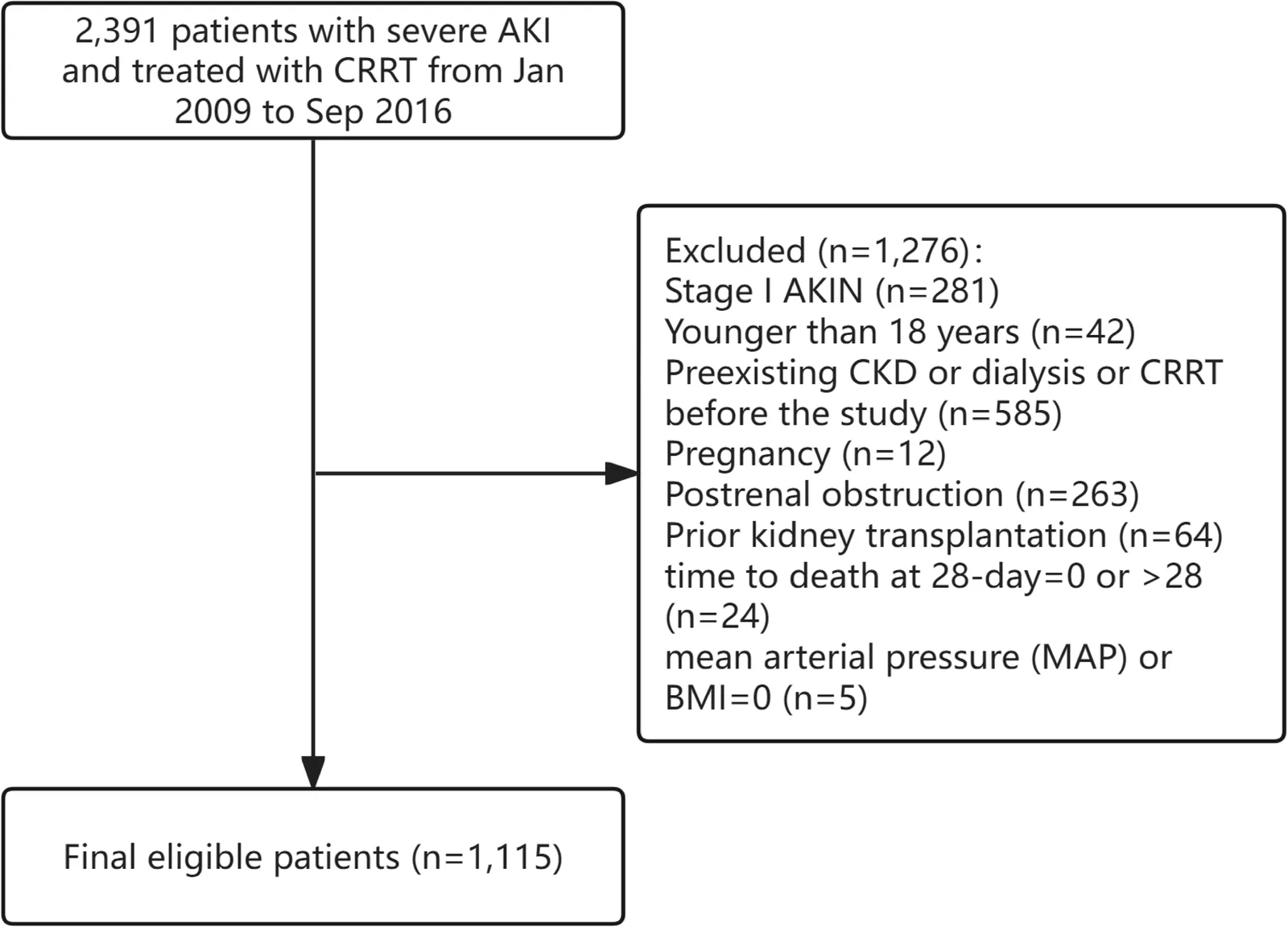
Flowchart of the patient selection process. AKIN = acute kidney injury network, BMI = body mass index, CCI = Charlson Comorbidity Index, CKD = chronic kidney disease.

As shown in Table 1, overall mortality following CRRT initiation in patients with severe AKI was 61.3% at 28 days and 71.1% at 90 days. Kaplan–Meier analysis demonstrated a graded decline in survival with increasing CCI (*P* for log-rank test < .0001, Fig. 2).

**Figure 2.**
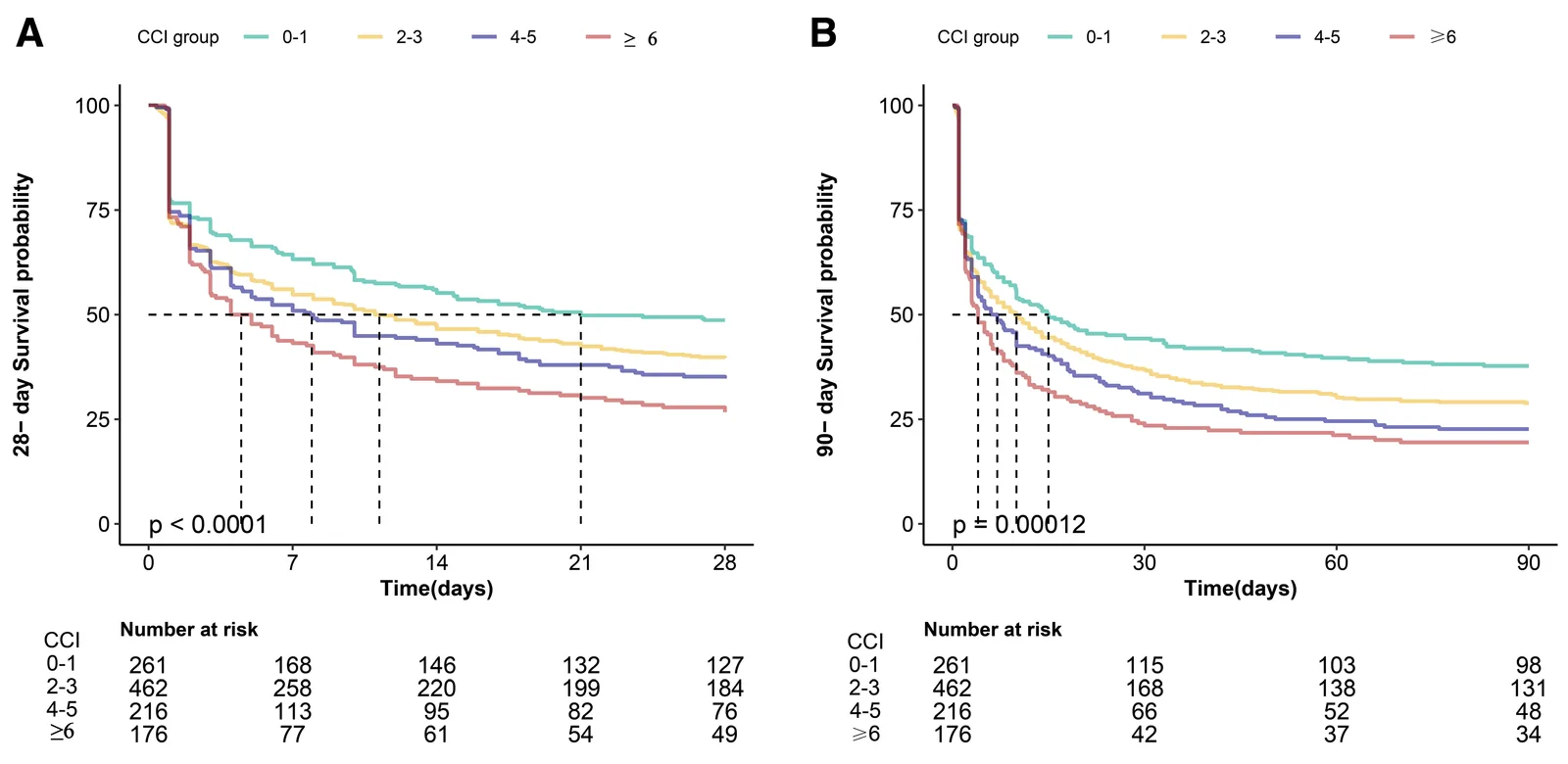
Kaplan–Meier estimates of 28- and 90-day survival according to CCI groups in patients with severe AKI. (A) 28-day mortality; (B) 90-day mortality. CCI = Charlson Comorbidity Index.

### 3.2. Association of CCI levels with 28- and 90-day mortality

This study evaluated the association between the CCI and 28- and 90-day mortality using Cox proportional hazards regression models. When CCI was a continuous predictor in the unadjusted Cox regression, CCI was associated with an increased hazard of death at 28 days (HR 1.07; 95% CI 1.04–1.11) and at 90 days (HR 1.06; 95% CI 1.03–1.10). After further adjustment for all potential covariates, associations remained significant, with an HR of 1.07 (95% CI, 1.03–1.11) and 1.06 (95% CI, 1.03‐1.09), respectively (*P* < .001, Table 2). As a categorized variable in the fully adjusted model, compared with the lowest CCI group (score = 0–1), the highest group (CCI ≥ 6) showed the largest hazard elevation (28-day HR 1.81, 95 % CI 1.40–2.33; 90-day HR 1.66, 1.31-2.10). A significant dose-response trend persisted across all covariate sets (*P* for trend < .001) (Table 2). Following multivariate adjustment, the CCI demonstrated a linear dose–response association with 28- and 90-day mortality (Supplementary Figure S1, Supplemental Digital Content 3).

**Table 2:** Association between CCI and 28-day and 90-day mortality in patients with severe AKI undergoing CRRT.

Variable	*n*.total	*n*.event%	Crude		Model1		Model2		Model3	
			HR (95% CI)	*P* value	HR (95% CI)	*P* value	HR (95% CI)	*P* value	HR (95% CI)	*P* value
28-d Mortality										
CCI	1115	683 (61.3)	1.07 (1.04~1.11)	<.001	1.08 (1.05~1.12)	<.001	1.08 (1.05~1.11)	<.001	1.07 (1.03~1.11)	<.001
CCI group										
0–1	261	134 (51.3)	Reference		Reference		Reference		Reference	
2–3	462	279 (60.4)	1.29 (1.05~1.59)	.015	1.39 (1.13~1.71)	.002	1.40 (1.14~1.73)	.002	1.40 (1.14~1.74)	.002
4–5	216	141 (65.3)	1.43 (1.13~1.81)	.003	1.56 (1.23~1.99)	<.001	1.64 (1.29~2.09)	<.001	1.40 (1.08~1.80)	.010
≥6	176	129 (73.3)	1.74 (1.37~2.22)	<.001	1.79 (1.40~2.29)	<.001	1.86 (1.45~2.38)	<.001	1.81 (1.40~2.33)	<.001
*P* for trend	1115	683 (61.3)		<.001		<.001		<.001		<.001
90-d Mortality										
CCI	1115	793 (71.1)	1.06 (1.03~1.10)	<.001	1.07 (1.03~1.10)	<.001	1.07 (1.04~1.10)	<.001	1.06 (1.03~1.09)	<.001
CCI group										
0–1	261	162 (62.1)	Reference		Reference		Reference		Reference	
2–3	462	325 (70.3)	1.24 (1.03~1.50)	.023	1.24 (1.03~1.50)	.025	1.31 (1.08~1.58)	.006	1.32 (1.09~1.61)	.005
4–5	216	165 (76.4)	1.41 (1.14~1.76)	.002	1.43 (1.15~1.78)	.001	1.52 (1.22~1.90)	<.001	1.40 (1.11~1.76)	.005
≥6	176	141 (80.1)	1.61 (1.28~2.02)	<.001	1.62 (1.29~2.03)	<.001	1.63 (1.29~2.05)	<.001	1.66 (1.31~2.10)	<.001
*P* for trend	1115	793 (71.1)		<.001		<.001		<.001		<.001

Crude Model: Non-adjusted.

Model 1: Adjusted for age, sex, and BMI.

Model 2: Adjusted for Model 1 + phosphate, creatinine, blood urea nitrogen, C-reactive protein, white blood cell count, hemoglobin, albumin, bicarbonate, potassium.

Model 3: Adjusted for Model 2 + 2h urine output at CRRT initiation, estimated glomerular filtration rate, mean arterial pressure, mechanical ventilation, APACHE II score, SOFA score, CRRT dose, cause of acute kidney injury, and AKI stages.

AKI = acute kidney injury, APACHE = acute physiology and chronic health evaluation, BMI = body mass index, CCI = Charlson comorbidity index, CI = confidence intervals, CRRT = continuous renal replacement therapy, HR = hazard ratio, SOFA = sequential organ failure assessment.

### 3.3. Sensitivity and subgroup analyses

Sensitivity and subgroup analyses were conducted to validate the main findings. Subgroup analyses were stratified by age (≥60 or <60 years), sex (male or female), SOFA score (<12 or 12), APACHE II score (<28 or ≥28), AKI stage (stage 2 or stage 3), and AKI cause (sepsis or non-sepsis). As shown in Figure 3, the trend in effect size was consistent across all subgroups. It was found that patients with sepsis showed an interaction between the CCI and 90-day mortality (*P* = .010). The *P*-value for the interaction in the other subgroups showed no interaction between the CCI and 28- or 90-day mortality.

**Figure 3.**
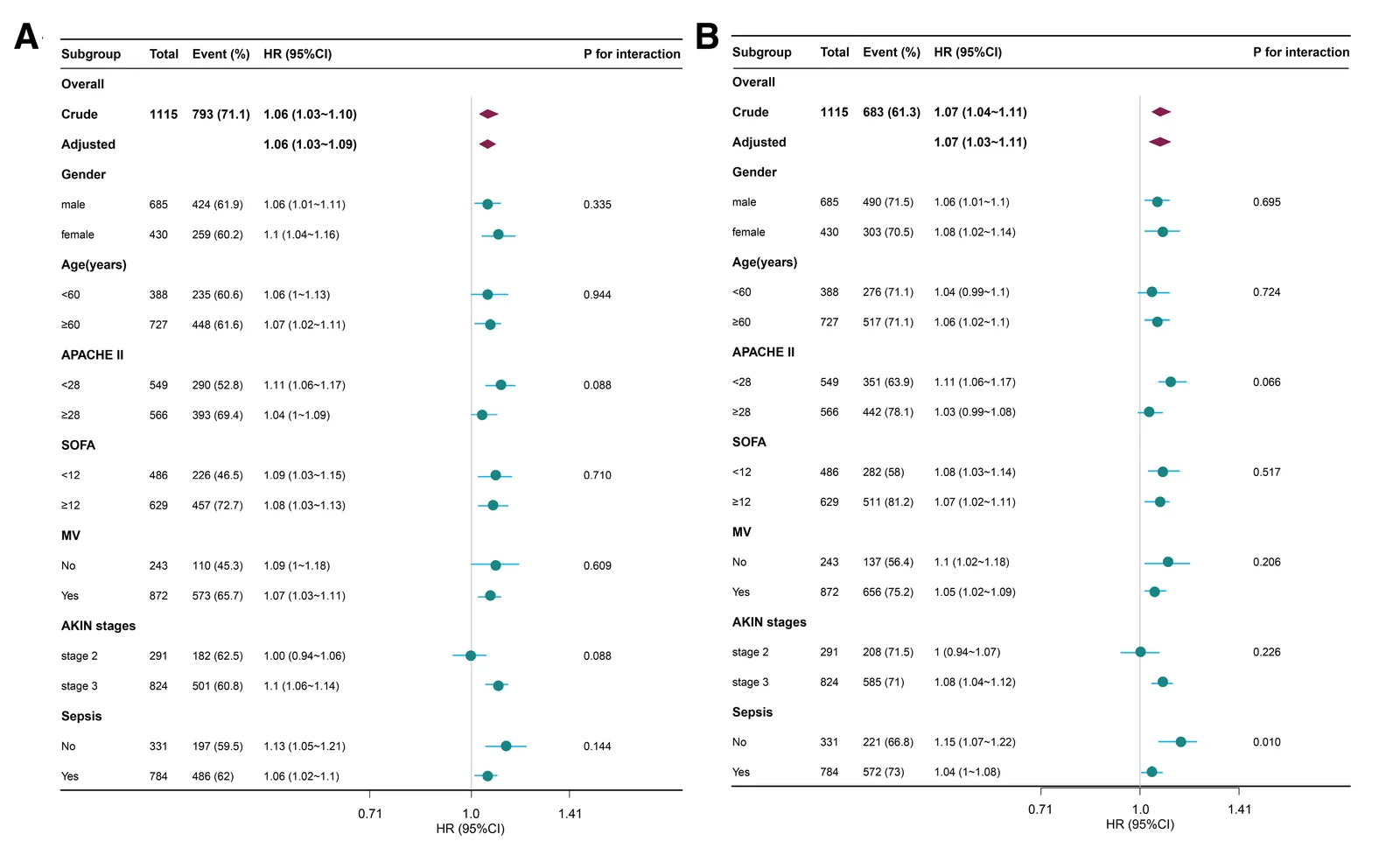
Forest plot for subgroup analysis of the association between CCI and mortality in severe AKI patients. (A) 28-day mortality; (B) 90-day mortality. Adjusted age, sex, BMI, phosphate, creatinine, blood urea nitrogen, C-reactive protein, white blood cell count, hemoglobin, albumin, bicarbonate, potassium, 2 hour urine output at CRRT initiation, estimated glomerular filtration rate, mean arterial pressure, mechanical ventilation, APACHE II score, SOFA score, CRRT dose, cause of acute kidney injury, and AKI stages. In each case, the model was not adjusted for the stratification variable. AKI = Acute kidney injury, APACHE = Acute Physiology and Chronic Health Evaluation, BMI = body mass index, CCI, = Charlson Comorbidity Index, CI = confidence interval, HR = hazard ratio, MAP = Mean arterial pressure, MV = Mechanical ventilation, SOFA = sequential organ failure assessment.

The CCI scores were divided into low and high groups based on the mean and median CCI scores of the participants. Using PSM, 369 pairs of patients were matched, and most covariates were balanced between the 2 groups (Supplementary Table S3, Supplemental Digital Content 4). The high CCI group had a significantly higher 28-day mortality (67.5% vs 57.2%, *P* < .001) and 90-day mortality (78.9% vs 66.1%, *P* < .001) (Supplementary Figure S2, Supplemental Digital Content 5). Furthermore, the association remained stable in the multivariate analysis using PSM, adjusted for propensity score, PA, and SMRW. The HRs were 1.25 to 1.36, all *P* < .05 (Table 3).

**Table 3:** Associations between CCI and the outcome in the crude analysis, multivariate analysis, and propensity score analyses.

Analysis	28-d mortality (%)	*P*	90-d mortality (%)	*P*
Events/ Total (%)				
CCI ≤ 3	413/723 (57.1)		487/723 (67.4)	
CCI > 3	270/392 (68.9)		306/392 (78.1)	
Crude analysis HR (95% CI)	1.32 (1.13~1.54)	<.001	1.30 (1.13~1.50)	<.001
Multivariate analysis HR (95% CI)	1.25 (1.06~1.47)	.007	1.25 (1.07~1.45)	.004
With matching	1.29 (1.08~1.55)	.006	1.36 (1.15~1.62)	<.001
With inverse probability weighting	1.26 (1.08~1.48)	.005	1.25 (1.08~1.45)	.004
Adjusted for propensity score	1.27 (1.08~1.49)	.004	1.27 (1.09~1.47)	.002
With SMRW	1.27 (1.09~1.48)	.004	1.27 (1.10~1.47)	.002
With PA	1.26 (1.05~1.51)	.004	1.27 (1.07~1.50)	.002

CCI = Charlson comorbidity index, CI = confidence interval, HR = hazard ratio, PA = pairwise algorithmic, SMRW = standardized mortality ratio weight.

### 3.4. Predictive ability of CCI for 28- and 90-day mortality

The predictive performance of the CCI, modeled as a continuous variable, for 28- and 90-day mortality was estimated ROC curves. C-statistics, NRI, and IDI were compared between the base and extended models that included the CCI (Figure 4, Table 4). The base model was comprised of the following baseline variables: age, BMI, SOFA and APACHE II scores, AKI stage and etiology, MV, phosphate level, creatinine level, and MAP. CCI inclusion significantly improved discrimination, with the C-statistic rising from 0.759 to 0.775 for 28-day mortality (*P* = .002) and from 0.763 to 0.777 for 90-day mortality (*P* = .017), and produced significant gains in NRI and IDI.

**Table 4:** Incremental prognostic value of CCI over base model for predicting 28- and 90-day mortality.

	C-statistics(95%CI)	IDI(95%CI)	NRI(95%CI)
28-d mortality			
Base	0.759 (0.740~0.788)	–	–
Base + CCI	0.775 (0.747~0.803)	0.010 (0.003~0.020)	0.097 (0.038~0.153)
*P* value	.011	<.001	.004
90-d mortality			
Base	0.763 (0.733~0.794)	–	–
Base + CCI	0.777 (0.748~0.807)	0.010 (0.003~0.020)	0.136 (0.052~0.199)
*P* value	.017	.004	<.001

The baseline model included age, BMI, SOFA score, APACHE II score, AKI stages, AKI causes, mechanical ventilation (MV), phosphate, creatinine, and mean arterial pressure (MAP).

CCI = Charlson comorbidity index, CI = confidence interval, IDI = integrated discrimination improvement, NRI = Net reclassification improvement.

**Figure 4.**
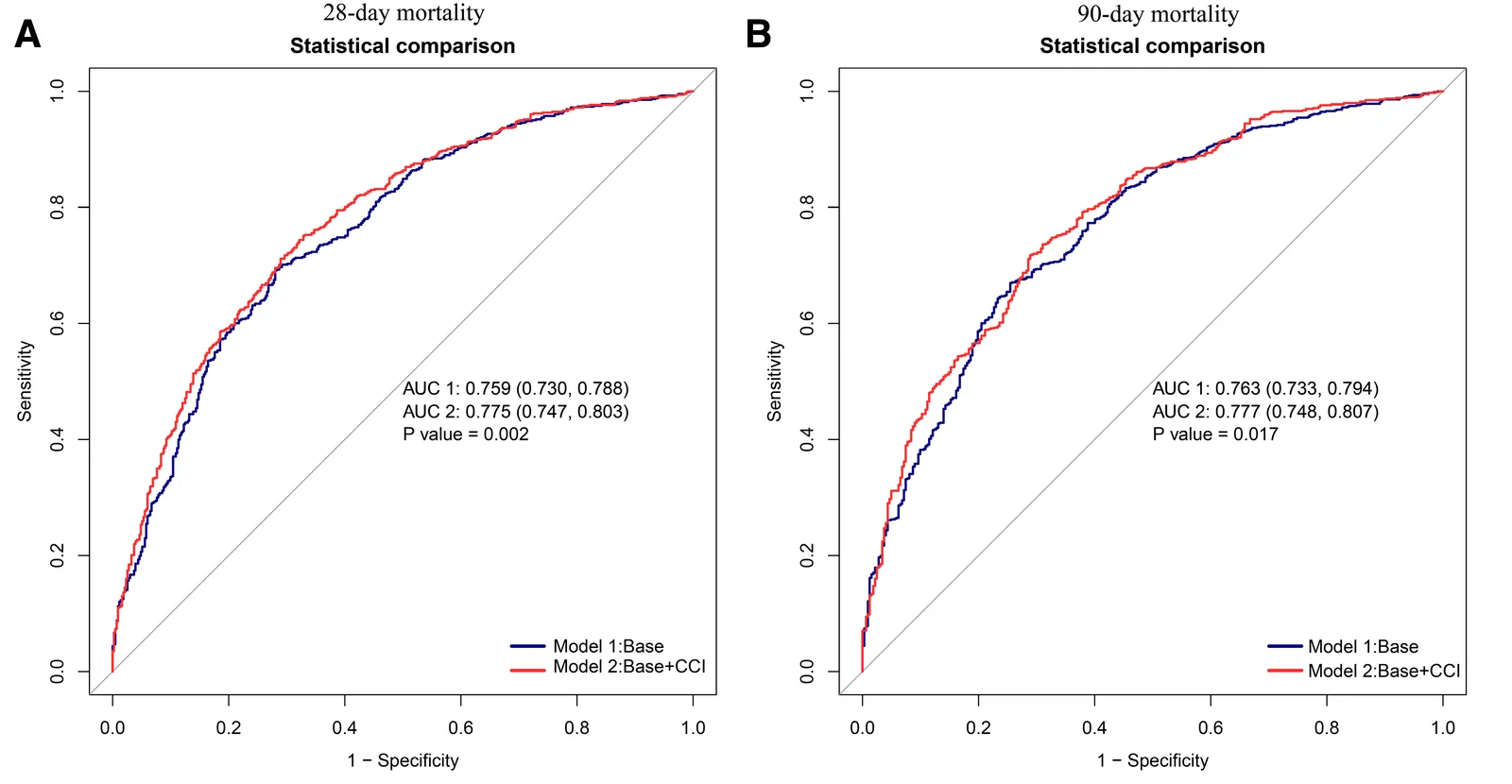
Receiver operating characteristic (ROC) curves assessing the predictive ability of CCI. (A) 28-day mortality; (B) 90-day mortality.Base model includes age, BMI, SOFA score, APACHE II score, AKI stages, AKI causes, MV, phosphate, creatinine, and MAP. Areas under the curve (AUC) are presented with 95 % confidence intervals. *P* values reflect the DeLong test for paired comparisons. AKI = acute kidney injury, APACHE = acute physiology and chronic health evaluation, BMI = body mass index, CCI = Charlson Comorbidity Index, MAP = mean arterial pressure, MV = mechanical ventilation, SOFA = sequential organ failure assessment.

## 4. Discussion

This study found that CCI was independently associated with both 28‑day and 90‑day mortality in patients with AKI undergoing CRRT. A higher CCI score was associated with a higher mortality rate. The association persisted after comprehensive adjustment for demographics, laboratory indices, organ support variables, illness severity (APACHE II, SOFA), and propensity score analyses. Furthermore, adding the CCI to a model of established risk factors may significantly enhance its predictive performance for short-term mortality.

These findings extend prior observations that the comorbidity burden predicts adverse outcomes in critically ill and AKI populations.^[8,21,22]^ The CCI has been widely validated as a predictor of short-term mortality across diverse inpatient cohorts.^[10,23–25]^ For instance, patients with a high CCI have an increased risk of all-cause mortality in those with delayed hemodialysis initiation.^[10]^ Similarly, Rodriguez et al found that the CCI was the sole predictor of mortality in patients with acute chest syndrome, reinforcing its role in risk stratification.^[24]^ Additionally, Leal et al highlighted that the CCI is an independent predictor of mortality risk after kidney transplant failure^.[23]^ Wang et al demonstrated that the CCI significantly contributes to a predictive model for 90-day mortality in patients with AKI undergoing CRRT, along with other clinical markers such as the SOFA score and SCr levels.^[25]^ Collectively, these findings underscore the additive value of comorbidity assessments in refining the prognostic models for high-risk populations. Few prior studies have focused specifically on CRRT cohorts or formally quantified incremental prognostic gains when comorbidity indices are added to established predictive models; by demonstrating both a graded hazard and measurable IDI/NRI improvements, our study extends these earlier findings and supports the complementary role of CCI in risk stratification.

There are several possible explanations for this. First, patients with multiple comorbidities may have problems with physiological reserves and impaired organ crosstalk resilience, making it more difficult to tolerate the systemic damage caused by severe AKI and CRRT.^[26]^ Second, common comorbidities (cardiovascular disease, diabetes, and chronic lung disease) are associated with chronic inflammation, endothelial dysfunction, and immune dysregulation, which may amplify acute injury, predispose patients to complications (e.g., infections and hemodynamic instability), and hinder renal and multi‑organ recovery.^[27–29]^

### 4.1. Strength of the study

Key strengths include a well‑characterized CRRT cohort, comprehensive adjustment for acute physiologic and biochemical variables, consistent findings across multivariate and PSM approaches, and formal assessment of incremental predictive value using contemporary metrics (C‑statistic, IDI, NRI). These design features supported the robustness and potential clinical relevance of the observed associations.

### 4.2. Limitation

This study has several limitations. The observational design cannot prove causality, and residual confounding remains possible, particularly from unmeasured constructs such as frailty phenotype, preadmission functional status, and variability in CRRT indications or practice. The cohort was derived from 2 tertiary hospitals, which may limit its generalizability to other settings. Finally, this study excluded baseline renal disease scoring, given the study population’s acute renal failure, which may have affected comparisons with studies that included chronic kidney disease as a comorbidity index.

## 5. Conclusions

In conclusion, a higher CCI independently predicted 28‑ and 90‑day mortality in patients with severe AKI receiving CRRT, adding prognostic value beyond the established acute severity scores. Routine CCI assessment may optimize early risk stratification and resource allocation in high-risk patients.

## Acknowledgements

We thank the collaborators and patients in the Dryad database. We appreciate the support of Dr Jie Liu of the Department of Vascular and Endovascular Surgery, Chinese PLA General Hospital, for assistance with study design, statistical support, and comments regarding the manuscript. We acknowledge the funding support from the Haidian District Health Development Scientific Research and Cultivation Plan Project (HP2025-98-507001).

## Author contributions

**Conceptualization:** Kejia Li, Yuefei Xiao.

**Formal analysis:** Kejia Li, Liqin Tang, Yunyun Yang.

**Methodology:** Kejia Li, Qingyan Ma, Jing Zhang.

**Writing – original draft:** Kejia Li, Jing Han.

**Writing – review & editing:** Yuefei Xiao, Chunhong Guo.










