# Supplementary material for: Association of Charlson comorbidity index with 28- and 90-day mortality in patients with severe acute kidney injury requiring CRRT: A retrospective cohort study
Source: Medicine (Baltimore). 2026 Sep 25;105(39):e50884. doi: 10.1097/MD.0000000000050884 (PMC13619215; doi:10.1097/MD.0000000000050884)
Supplement: Supplementary file 1 [file medi-105-e50884-s001.docx]

**Supplementary Table S1. Charlson Comorbidity Index**

| Score | Comorbid condition |
| --- | --- |
| 1 | Myocardial infarction  Congestive heart failure  Cerebral vascular disease  Peripheral vascular disease  Dementia  Chronic obstructive pulmonary disease  Connective tissue disease  Peptic ulcer disease  Mild liver disease  Diabetes |
| 2 | Hemiplegia  Diabetes with end-organ damage  Any solid tumor  Leukemia  Lymphoma |
| 3 | Moderate/severe liver disease |
| 6 | Metastatic solid tumor  Acquired immunodeficiency syndrome |
