# Supplementary material for: Association of Charlson comorbidity index with 28- and 90-day mortality in patients with severe acute kidney injury requiring CRRT: A retrospective cohort study
Source: Medicine (Baltimore). 2026 Sep 25;105(39):e50884. doi: 10.1097/MD.0000000000050884 (PMC13619215; doi:10.1097/MD.0000000000050884)
Supplement: Supplementary file 2 [file medi-105-e50884-s002.docx]

**Supplementary Table S2. Covariate screening of model altered the effect size by at least 10%**

| Term1 | β1 | Change.  percentage1 | Term2 | β2 | Change.  percentage2 | GVIF | DF | GVIF^  (1/(2*Df)) | colinearity | select | select.VIF |
| --- | --- | --- | --- | --- | --- | --- | --- | --- | --- | --- | --- |
| Crude | 0.06 | Ref. | Full | 0.01 | Ref. | 1.178 | 1 | 1.086 | 0 | Ref. | Ref. |
| Age | 0.06 | 1.1 | Age | 0.01 | 5.8 | 1.468 | 1 | 1.212 | 0 | No | No |
| Sex | 0.06 | 0.8 | Sex | 0.01 | -21.2 | 1.363 | 1 | 1.168 | 0 | Yes | Yes |
| Potassium | 0.07 | 5.2 | Potassium | 0.01 | 26.4 | 1.579 | 1 | 1.256 | 0 | Yes | Yes |
| Bicarbonate | 0.07 | 4.0 | Bicarbonate | 0.01 | -12.3 | 1.663 | 1 | 1.290 | 0 | Yes | Yes |
| Phosphate_0h | 0.06 | 1.3 | Phosphate_0h | 0.01 | 7.7 | 49.603 | 1 | 7.043 | 1 | No | Pending |
| Phosphate_24h | 0.05 | -19.8 | Phosphate_24h | 0.01 | 0.5 | 64.474 | 1 | 8.030 | 1 | Yes | Pending |
| Delta_Phosphate | 0.06 | -5.4 | Delta_Phosphate | 0.01 | 18.2 | 55.74 | 1 | 7.466 | 1 | Yes | Pending |
| BMI | 0.06 | -0.8 | BMI | 0.01 | -11.4 | 1.144 | 1 | 1.070 | 0 | Yes | Yes |
| SBP | 0.07 | 10.3 | SBP | 0.01 | 13.7 | 25.802 | 1 | 5.080 | 1 | Yes | Pending |
| DBP | 0.07 | 5.9 | DBP | 0.01 | 0.3 | 42.274 | 1 | 6.502 | 1 | No | Pending |
| MAP | 0.07 | 11.6 | MAP | 0.01 | 2.0 | 96.100 | 1 | 9.803 | 1 | Yes | Pending |
| MV | 0.07 | 17.6 | MV | 0.01 | 39.6 | 2.023 | 1 | 1.422 | 0 | Yes | Yes |
| White blood cell | 0.06 | -1.7 | White blood cell | 0.01 | -1.6 | 1.130 | 1 | 1.063 | 0 | No | No |
| Haemoglobin | 0.06 | -8.7 | Haemoglobin | 0.01 | 12.6 | 1.302 | 1 | 1.141 | 0 | Yes | Yes |
| BUN | 0.06 | -3.5 | BUN | 0 | -48.9 | 1.884 | 1 | 1.372 | 0 | Yes | Yes |
| Creatinine | 0.07 | 10.9 | Creatinine | 0 | -145.5 | 3.577 | 1 | 1.891 | 0 | Yes | Yes |
| Albuminh | 0.06 | 0.7 | Albuminh | 0 | -33.4 | 1.408 | 1 | 1.187 | 0 | Yes | Yes |
| CRP | 0.05 | -19.6 | CRP | 0.03 | 259.6 | 1.276 | 1 | 1.130 | 0 | Yes | Yes |
| eGFR | 0.07 | 5.1 | eGFR | 0.01 | 4.4 | 2.973 | 1 | 1.724 | 0 | No | No |
| Urine output in 2h | 0.06 | -1.1 | Urine output in 2h | 0.01 | 18.3 | 1.155 | 1 | 1.075 | 0 | Yes | Yes |
| APACHE II score | 0.06 | -0.9 | APACHE II score | 0 | -63 | 1.571 | 1 | 1.253 | 0 | Yes | Yes |
| SOFA score | 0.05 | -16.1 | SOFA score | 0.02 | 212.0 | 1.784 | 1 | 1.336 | 0 | Yes | Yes |
| CRRT dose | 0.06 | -0.4 | CRRT dose | 0.01 | 5.9 | 1.174 | 1 | 1.084 | 0 | No | No |
| AKIN | 0.06 | -0.1 | AKIN | 0.01 | 23.7 | 1.496 | 1 | 1.223 | 0 | Yes | Yes |
| AKI cause | 0.06 | 3.5 | AKI cause | 0 | -93.1 | 1.914 | 4 | 1.085 | 0 | Yes | Yes |

**Abbreviations:** CCI, Charlson Comorbidity Index; BMI, Body mass index; BUN, Blood urea nitrogen; CRP, C-reactive protein; SBP, Systolic blood pressure; DBP, Diastolic blood pressure; MAP, Mean arterial pressure; MV, Mechanical ventilation; eGFR, Estimated glomerular filtration rate; APACHE, Acute Physiology and Chronic Health Evaluation; SOFA, Sequential Organ Failure Assessment; CRRT, Continuous renal replacement therapy; AKI, Acute kidney injury.

No multicollinearity was detected between CCI and other variables. However, the variables whose effect size changed by less than 10% when included in the overall model were age, Phosphate_0h, Phosphate_24h, DBP, MAP, white blood cell, eGFR, and CRRT dose.
