# Supplementary material for: Association of Charlson comorbidity index with 28- and 90-day mortality in patients with severe acute kidney injury requiring CRRT: A retrospective cohort study
Source: Medicine (Baltimore). 2026 Sep 25;105(39):e50884. doi: 10.1097/MD.0000000000050884 (PMC13619215; doi:10.1097/MD.0000000000050884)
Supplement: Supplementary file 3 [file medi-105-e50884-s004.docx]

**Supplementary Table S3. Imbalance of patient characteristics before and after propensity score matching**

| Item | before PSM | | | | after PSM | | | |
| --- | --- | --- | --- | --- | --- | --- | --- | --- |
|  | CCI≤3 (n=723) | CCI>3 (n=392) | SMD | SMD  -0.1 | CCI≤3  (n=369) | CCI>3  (n=369) | SMD | SMD  -0.1 |
| Age( years) | 63.45 (14.73) | 63.23 (13.48) | 0.016 | <0.1 | 62.88 (14.47) | 63.16 (13.39) | 0.020 | <0.1 |
| Female,n(%) | 297 (41.10) | 133 (33.90) | 0.148 | >0.1 | 121 (32.80) | 128 (34.70) | 0.040 | <0.1 |
| BMI (kg/m²) | 23.78 (4.36) | 23.98 (4.43) | 0.045 | <0.1 | 23.94 (4.30) | 23.84 (3.94) | 0.023 | <0.1 |
| Potassium (mEq/L) | 4.70 (1.06) | 4.69 (1.12) | 0.010 | <0.1 | 4.69 (1.05) | 4.68 (1.11) | 0.012 | <0.1 |
| Bicarbonate (mEq/L) | 17.06 (5.63) | 16.72 (6.05) | 0.058 | <0.1 | 16.31 (5.71) | 16.93 (6.04) | 0.106 | >0.1 |
| Phosphate (mg/dL) | 5.65 (2.50) | 5.84 (2.18) | 0.082 | <0.1 | 5.93 (2.68) | 5.82 (2.19) | 0.046 | <0.1 |
| White blood cell (×10³µL) | 14.16 (13.86) | 14.21 (11.58) | 0.005 | <0.1 | 14.59 (16.96) | 14.18 (11.54) | 0.029 | <0.1 |
| Haemoglobin (g/dL) | 9.83 (2.26) | 9.34 (2.06) | 0.224 | >0.1 | 9.41 (2.18) | 9.41 (2.08) | <0.001 | <0.1 |
| BUN ( mg/dL) | 53.41 (29.45) | 60.32 (30.62) | 0.230 | >0.1 | 57.74 (32.87) | 58.88 (29.73) | 0.036 | <0.1 |
| Creatinine (mg/dL) | 2.62 (1.52) | 2.97 (1.79) | 0.212 | >0.1 | 2.91 (1.71) | 2.93 (1.78) | 0.011 | <0.1 |
| Albumin (g/dL) | 2.62 (0.59) | 2.63 (0.54) | 0.009 | <0.1 | 2.62 (0.59) | 2.63 (0.54) | 0.015 | <0.1 |
| CRP (mg/L) | 109.37 (106.22) | 108.63 (112.37) | 0.007 | <0.1 | 117.62 (112.70) | 108.83 (113.72) | 0.078 | <0.1 |
| MAP (mmHg) | 77.36 (14.23) | 78.51 (13.98) | 0.081 | <0.1 | 78.49 (14.77) | 78.28 (14.13) | 0.015 | <0.1 |
| MV, n (%) | 586 (81.10) | 286 (73.00) | 0.193 | >0.1 | 273 (74.00) | 279 (75.60) | 0.037 | <0.1 |
| eGFR (mL/min/1.73m²) | 32.55 (22.77) | 28.65 (17.91) | 0.190 | >0.1 | 30.29 (23.11) | 29.14 (18.08) | 0.056 | <0.1 |
| Urine output in 2h (mL) | 78.01 (103.50) | 64.27 (101.70) | 0.134 | >0.1 | 76.81 (99.46) | 65.84 (103.20) | 0.108 | >0.1 |
| APACHE II score | 27.30 (7.89) | 27.08 (8.06) | 0.027 | <0.1 | 27.24 (8.29) | 27.28 (8.00) | 0.005 | <0.1 |
| SOFA score | 11.80 (3.33) | 12.47 (3.86) | 0.184 | >0.1 | 12.40 (3.30) | 12.47 (3.90) | 0.019 | <0.1 |
| CRRT dose (mL/kg) | 36.78 (4.73) | 36.48 (4.86) | 0.062 | <0.1 | 36.41 (4.82) | 36.56 (4.89) | 0.032 | <0.1 |
| AKI stage, n (%) | 533 (73.70) | 291 (74.20) | 0.012 | <0.1 | 270 (73.20) | 275 (74.50) | 0.031 | <0.1 |
| Sepsis, n (%) | 528 (73.00) | 256 (65.30) | 0.168 | >0.1 | 244 (66.10) | 243 (65.90) | 0.006 | <0.1 |

**Abbreviations:** CCI, Charlson Comorbidity Index; BMI Body mass index; BUN, Blood urea nitrogen; CRP, C-reactive protein; MAP, Mean arterial pressure; MV, Mechanical ventilation; eGFR, Estimated glomerular filtration rate; APACHE, Acute Physiology and Chronic Health Evaluation; SOFA, Sequential Organ Failure Assessment; CRRT, Continuous renal replacement therapy;AKI, Acute kidney injury, SMD, Standardized mean differences.

**Notes:** An absolute SMD<10% was considered to support the assumption of a balance between the groups. Data are presented as medians [interquartile ranges], mean [SD] or as numbers (percentages).
